# Allergic proctocolitis refractory to maternal hypoallergenic diet in exclusively breast-fed infants: a clinical observation

**DOI:** 10.1186/1471-230X-11-82

**Published:** 2011-07-16

**Authors:** Sandra Lucarelli, Giovanni Di Nardo, Ginevra Lastrucci, Ylenia D'Alfonso, Adriana Marcheggiano, Tatiana Federici, Simone Frediani, Tullio Frediani, Salvatore Cucchiara

**Affiliations:** 1 Pediatric Gastroenterology Endoscopy and Liver Unit, Sapienza University of Rome, Azienda Policlinico Umberto I, Viale Regina Elena 324, 00161 Rome, Italy; 2 Pediatric Allergology Unit, Sapienza University of Rome, Azienda Policlinico Umberto I, Viale Regina Elena 324, 00161 Rome, Italy; 3 Department of Clinical Sciences, Sapienza University of Rome, Azienda Policlinico Umberto I, Viale Regina Elena 324, 00161 Rome, Italy

**Keywords:** allergic proctocolitis, food allergy, breast feeding, patch test

## Abstract

**Background:**

Allergic proctocolitis (APC) in exclusively breast-fed infants is caused by food proteins, deriving from maternal diet, transferred through lactation. In most cases a maternal cow milk-free diet leads to a prompt resolution of rectal bleeding, while in some patients a multiple food allergy can occur. The aim of this study was to assess whether the atopy patch test (APT) could be helpful to identify this subgroup of patients requiring to discontinue breast-feeding due to polisensitization. Additionally, we assessed the efficacy of an amino acid-based formula (AAF) when multiple food allergy is suspected. amino acid-based formula

**Methods:**

We have prospectively enrolled 14 exclusively breast-fed infants with APC refractory to maternal allergen avoidance. The diagnosis was confirmed by endoscopy with biopsies. Skin prick tests and serum specific IgE for common foods, together with APTs for common foods plus breast milk, were performed. After a 1 month therapy of an AAF all patients underwent a follow-up rectosigmoidoscopy.

**Results:**

Prick tests and serum specific IgE were negative. APTs were positive in 100% infants, with a multiple positivity in 50%. Sensitization was found for breast milk in 100%, cow's milk (50%), soy (28%), egg (21%), rice (14%), wheat (7%). Follow-up rectosigmoidoscopy confirmed the remission of APC in all infants.

**Conclusions:**

These data suggest that APT might become a useful tool to identify subgroups of infants with multiple gastrointestinal food allergy involving a delayed immunogenic mechanism, with the aim to avoid unnecessary maternal dietary restrictions before discontinuing breast-feeding.

## Background

Allergic proctocolitis (APC) is a cause of rectal bleeding in exclusively breast-fed infants aged from 1 to 6 months [1]. It is due, in most cases, to cow's milk proteins transferred via breast milk [2]. Diagnosis is based on clinical features and recovery after dietetic therapy [3]. Rectal bleeding generally resolves within 72-96 hours of cow's milk (CM) protein maternal avoidance. However, it is reported that in about 7% of cases an extensively hydrolyzed formula (eHF) must be used, and 5% require an amino acid-based formula (AAF) [1]. Endoscopic biopsies show superficial erosions, eosinophilic infiltration and frequent lymphoid nodular hyperplasia (LNH) [4-6]. Prick tests and serum specific IgE are typically negative, indeed a non-IgE mediated pathogenetic mechanism is thought to be involved; currently there are no available data on the use of the atopy patch test (APT) in infants with APC [5]. Present study reports a series of 14 exclusively breast fed infants with histologically documented APC and persistent bleeding refractory to maternal dietary restrictions. The aim of our study was to evaluate whether APT could detect non-IgE-mediated polisensitization to food proteins carried via breast milk; additionally, we assessed the efficacy of AAF when multiple food allergy is suspected.

## Methods

The study was approved by the Ethical Committee of Research of the University "La Sapienza" in Rome (protocol number 343/07 of 26/04/07), and written informed consent was obtained from parents of all patients. This prospective research study was carried out at the Paediatric Gastroenterology Unit of Umberto I Hospital in Rome from 2007 to 2010. We consecutively enrolled 14 exclusively breast-fed infants (10 M: 4 F), aged 15 days to 6 months, with haematochezia due to likely APC, which did not resolve with an oligoantigenic maternal diet. The main symptom present in all patients was rectal bleeding, variably associated to mucus in stools (64%), chronic diarrhea (42%), and minor gastrointestinal complaints such as gastro-esophageal reflux disease (21%) and colics (21%). A family history of atopy was present in 64%. On admission, all patients were exclusively breast fed, and a CM-free diet was previously administered by pediatricians to mothers; also soy, egg, or both, were empirically eliminated. The duration of this hypoallergenic diet varied from 1 to 9 weeks (mean 4 ± 2 weeks) (Table 1).

**Table 1:** Major features of the 14 exclusively breast-fed infants with allergic proctocolitis

Patient (sex)	Clinical Onset		Maternal elimination. diet (*)		Our observation		Atopy patch test	Endoscopic features		Histology Eosinophiles 10HPF (40x)		Current status (age)
	**Age (days)**	**Symptoms**	**Allergen avoidance**	**Duration**	**Age (days)**	**Symptoms**		**Baseline**	Follow-up (**)	**Baseline**	Follow-up (**)	

1 (F)	60	Haematochezia; GERD	CM S	4 weeks	90	Severe rectal bleeding; syderopenic anemia	BM ++++ CM +	Scattered erosions; ileo-colonic LNH	Normal mucosa	> 60	Normal features	Free diet, well (by 3 years)

2 (M)	90	Haematochezia; GERD	CM E S	8 weeks	180	Melena; hematemesis	BM +++ CM ++	Diffuse erosions; ileal LNH	Mild LNH	> 60	Normal features	Free diet, well (by 2 years)

3 (M)	120	Chronic diarrhea	CM E	6 weeks	160	Severe rectal bleeding	BM ++ S +	Scattered erosions; ileo-colonic LNH	Mild LNH	> 60	Normal features	Free diet, well (by 2 years)

4 (M)	10	Blood and mucus in stools	CM E S	3 weeks	30	Hematochezia	BM +++ CM + E +	Friability; initial LNH	Normal mucosa	40-50	Normal features	CM and E-free diet (13 months)

5 (M)	20	Bloody diarrhea	CM	1 week	30	Bloody diarrhea; syderopenic anemia	BM ++	Scattered erosions; initial LNH	Normal mucosa	40-50	Normal features	Free diet, well (by 15 months)

6 (F)	20	Hematochezia; GERD	CM S	9 weeks	120	Diarrhea; mucus in stools	BM +++	Diffuse LNH	Mild LNH	> 60	Normal features	CM and E-free diet (11 months)

7 (M)	7	Fluid diarrhoea and feeding refusal	CM E	1 week	15	Hematochezia	CM ++ R +	Rectal erosions	Normal mucosa	40-50	Normal features	CM and R-free diet (7 months)

8 (M)	180	Haematochezia	CM E S	2 weeks	195	Severe rectal bleeding; dehydration	BM + CM ++ E ++ S ++ W + R ++	Diffuse LNH	Normal mucosa	> 60	Normal features	CM, S, E and R-free diet (15 months)

9 (F)	40	Blood and mucus in stools	CM E	3 weeks	60	Hematochezia	BM ++++	Hedema; hyperhemia; scattered erosions	Normal mucosa	40-50	Normal features	CM and E-free diet (16 months)

10 (M)	28	Blood and mucus in stools; colics	CM E S	4 weeks	60	Bloody diarrhea; mucus in stools	BM ++ S ++	Diffuse erosions; ileo-colonic LNH	Normal mucosa	> 60	Normal features	Free diet, well (by 1 year)

11 (M)	30	Blood and mucus in stools; colics	CM S	4 weeks	60	Severe rectal bleeding; colics	BM +++ CM +	Scattered erosions; friability; ileo-colonic LNH	Normal mucosa	40-50	Normal features	Free diet, well (by 1 year)

12 (F)	45	Diarrhea with mucus and blood; irritability	CM E S	6 weeks	60	Severe rectal bleeding	BM +++ CM + S +	Scattered erosions; ileo-colonic LNH	Mild LNH	40-50	Normal features	Free diet, well (by 1 year)

13 (M)	20	Blood and mucus in stools; colics	CM	1 week	30	Bloody diarrhea; mucus in stools	BM + E +	Scattered erosions; friability ileo-colonic LNH	Normal mucosa	> 60	Normal features	CM and E-free diet (14 months)

14 (M)	21	Diarrhea with mucus and blood; irritability	CM E S	1 week	30	Bloody diarrhea; irritability	BM++	Diffuse erosions; ileal LNH	Normal mucosa	> 60	Normal features	Free diet, well (by 1 year)

(*) the diet was empirically administered by pediatricians before our observation; (**) after 30 days of exclusively elimination diet with amino acid-based formula; GERD = gastroesophageal reflux disease; HPF = high power field; LNH = lymphoid nodular hyperplasia; CM = cow's milk; E = egg; S = soy; W = wheat; R = rice; BM = breast milk.

### Clinical and laboratory examination

All infants underwent clinical and auxological evaluation, routine biochemistry, parassitological and coltural stools studies for common pathogens.

### Determination of serum specific IgE levels

Concentrations of serum specific IgE antibody titers to common foods (CM, soy, rice, wheat, egg) were measured using the immuno-CAP system with a detection limit of 0,35 kU/L IgE [7].

### Skin prick tests

Prick tests for common food proteins (CM, soy, rice, wheat, egg) were performed on the forearm with a lancet with a 1 mm tip (Lofarma^®^, Milano, Italy) using commercial extracts (Lofarma^®^, Milano, Italy). In each instance, we pricked through a drop of the extract which was then absorbed; positive histamine and negative diluent controls were used. We recorded the largest diameter of the wheal (in millimeters) at 15 minutes; a wheal of 3 mm greater than the negative control was considered positive reaction [8].

### Atopy patch test (APT)

APTs for common food proteins (CM, soy, rice, wheat, egg) and for maternal breast milk were done. On the test day, 2 g of fresh foods (cow's milk, soy formula, rice cream, egg) or dry food mixed with 2 mL isotonic saline solution (wheat flour), and 2 mL of maternal breast milk, were placed on Large Finn chambers on Scanpor (Haye's Service^®^, The Netherlands) [9]. A negative (petrolatum) vehicle control was applied with each APT. The patch adhered to the patient's back and was removed at 48 hours; the results were read after 20 minutes and at 72 hours [10]. According to ETFAD consensus, APT reactions were graded as follows: + (erythema plus slight infiltration); ++ (erythema plus ≤ 3 papules); +++ (erythema plus > 4 papules); ++++ (erythema plus vescicles [11].

### Endoscopy and histology

Patients underwent an ileo-colonoscopy (or rectosigmoidoscopy in 4 patients, according to age) to confirm the diagnosis and to exclude other sources of rectal bleeding. Endoscopic evaluation was performed by one pediatric gastroenterologist with neonatal videocolonoscopes (Olympus^®^), after deep sedation with propofol (induction dose: 1 to 2 mg/kg; repeated dose: 0.5 to 1 mg/kg) or a mild sedation with midazolam (0.2 mg/kg) prior to ileo-colonoscopy or rectosigmoidoscopy, respectively. Pathological findings were noted and recorded by photos. Biopsy specimens were taken from the terminal ileum, from each segment of the colon, and from areas where lesions were noted. Histological examination was performed by a pathologist unaware of the clinical and laboratory data of the patients.

### Dietetic therapy and Follow-up

Breast feeding was discontinued and exclusive feeding with an AAF was started. After a one-month elemental diet, patients underwent a follow-up rectosigmoidoscopy. If rectal bleeding stopped and mucosal healing was confirmed, the elemental diet continued for 4 to 6 months, based on age. An oligoantigenic diet based on lamb and rice or maize and olive oil [12] was introduced for a period of 2 months. Subsequently, they underwent open food challenges to assess the development of tolerance to food allergens: vegetables, fruits and grains at 6 months; beef and poultry at 7 months; cow's milk proteins, eggs, fish, and tropical fruits at 12 months. Nuts and peanuts were always excluded until the child was older than 3 years. Additional foods could be challenged every 15 days if no delayed reaction occurred. Infants had a monthly or bi-monthly clinical follow-up until they were able to tolerate a free diet.

## Results

**Figure 1:**
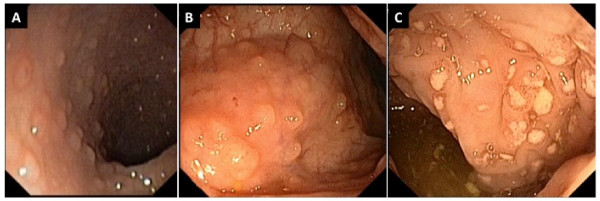
**Endoscopic findings showing colonic (A), ileal LNH(B) and rectal aphtoid ulcers (C) in a child with dietary protein-induced allergic proctocolitis**.

**Figure 2:**
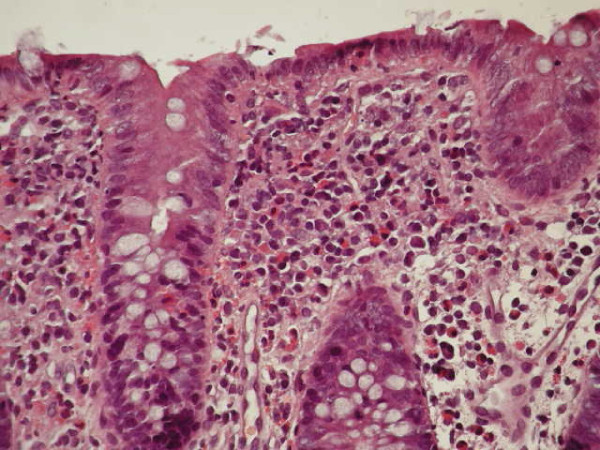
**Significant (> 60/10 HPF) eosinophilic infiltration of the colonic mucosa in a child with dietary protein-induced allergic proctocolitis**.

The clinical, allergological and endoscopical features of the 14 infants are summarized in Table 1. Clinical examination was unremarkable except for abdominal swelling; growth was good in all but two infants. The laboratory findings showed sideropenic anemia in two patients, peripheral eosinophilia in one, and mild hypertransaminasemia in two. Prick tests and serum specific IgE for foods were negative. The APTs were positive for breast milk in all infants, CM in 50%, soy in 28%, egg in 21%, rice in 14% and wheat in 7%; a multiple positivity was recorded in 50%. Endoscopy showed widespread hyperhaemic and edematous rectal mucosa with microerosions in 21%, erythema with scattered rectosigmoid aftoid erosions in 50%, and diffuse colonic hyperhaemic and edematous mucosa in 35%. LNH was present in the terminal ileum, left colon and rectum in 42%, in the terminal ileum, colon and rectum in 14%, and exclusively in the terminal ileum in 14% (Figure 1). LNH was defined as a cluster of > 10 extruding lymphoid nodules, as previously described [13]. The diagnosis of APC was confirmed by the presence of a large number of eosinophils in the lamina propria in at least one of the biopsy specimens collected [6]. In 43% infants, ≥ 60 eosinophils × 10 high power field (HPF) were found (Figure 2), while in the remaining a lower number of eosinophils (40-50 × 10 HPF) was observed. Non-specific signs of inflammation, such as edema and lymphoplasmocitic infiltrate, were also present, and the overall architecture of the mucosa was always conserved.

The infants showed progressive resolution of rectal bleeding within 72 hours of exclusive feeding with AAF, and were discharged in good general condition. The follow-up rectosigmoidoscopy showed complete normalization of mucosa in all patients; only a mild nodularity persisted in 50% of infants with colonic LNH. The oligoantigenic diet was well tolerated; all food was successfully introduced at 15 months of age in five patients, at 2 years in two, and at 3 years in one, while currently 6 patients are still on a hypoallergenic diet.

## Discussion

APT positivity for foods and breast milk, in our case series of exclusively breast-fed infants with APC, confirms the hypothesis that food proteins can be transferred through lactation and are potentially able to induce an allergic reaction in infants [14-19]. Indeed, more than 50% of cases of APC reported in literature are exclusively breast-fed infants, and in most cases a gradual and complete resolution of the disease can be observed after 72-96 hours of maternal avoidance of offending proteins [1-4,6,14-18]. Lake reported the most common causative food is cow's milk (65%), but also egg, corn and soy can be implicated (in 19%, 6%, 3%, respectively) and about 5% of infants have an identified multiple food allergy [1]. In our case series, APTs for foods detected a sensitization to CM in 50%, soy in 28%, egg in 21%, rice in 14%, wheat in 7%, thus suggesting that CM is probably the most common causative food in infants with APC. However, note that the APT negativity for foods eliminated from the maternal diet, such as CM, might also be due to the long period of allergen avoidance with consequent desensitization of infant. Furthermore, the positivity in all patients of APTs for breast milk of mothers on hypoallergenic (CM plus soy and/or egg-free) diets is strongly suggestive of the involvement of many allergens other than CM.

It is reported in about 12% of cases, offending foods could not be identified through maternal dietary manipulation and breast feeding maintenance led to intermittent persistent bleeding [1]. Similarly, our patients represent that select subgroup of infants which require discontinuing breast feeding; we speculate their non-responsiveness could be due to a multiple food allergy, as shown by both the polisensitization detected by APTs in 50% of patients and the APTs positivity for BM of mothers on hypoallergenic diet. In those requiring hypoallergenic formulas, about 41% could need an AAF due to non-responsivity to extensively Hydrolized Formula (eHF). Our choice to administer an AAF, prior to trying an eHF as guidelines would recommend [20], was based on the potential allergenicity of eHF, due to residual immunologically active proteins, in highly allergic children [21-23]. This is particularly an issue for children with allergy that is not directly related to food-specific IgE antibody, such as APC. Probably the gastrointestinal allergic reactions are more likely to be triggered by residual peptides in eHF because T-cell epitopes are typically smaller than B-cell, IgE-binding, epitopes [24]. Children with gastrointestinal food allergy, which are presumably T-cell-mediated, may therefore be more likely to react to eHF, thus representing a group for whom AAF is particularly important, as in our case series.

The diagnosis of APC is commonly based on clinical features and the response to maternal avoidance of the offending proteins. In refractory cases, we suggest to perform endoscopical examination with the aim to exclude any possible cause of rectal bleeding, other than APC, which might explain symptom persistence [2]. The clinical suspicion of APC was confirmed in our patients by colonoscopic features and biopsies, which showed eosinophilic inflammation and LNH, which is a common finding in food allergy [13,25,26]. After one month of elemental feeding LNH significantly decreased or completely disappeared and colonic mucosa was normal. Positive response to the AAF also suggests the presence of food allergy as the possible cause of gastrointestinal complaints. However, in our case series, the diagnostic oral provocation test was not performed for ethical considerations due to the severity of clinical findings. Rectosigmoidoscopic follow-up was considered more ethically correct to confirm mucosal healing.

To the best of our knowledge, no data is currently available on the use of APT in infants with APC. Many studies shown the ability of APT in detecting delayed food reactions in infants with atopic dermatitis, with a high specificity and poor sensitivity [27,28]. Together with prick testing and serum specific IgE assay, a positive predictive value of about 95% can be attained [29]. Its usefulness was also proposed in the diagnostic work-up of non-IgE-mediated gastrointestinal food allergy in patients with growth distrurbance, rectal bleeding and gastro-esophageal reflux disease [30,31], eosinophilic esophagitis [32] and food protein-induced enterocolitis syndrome [33]. Positive APT, together with negative prick tests and negative serum specific IgE, suggest food proteins carried via breast milk can sensitize exclusively breast-fed infants, and then trigger a T lymphocyte-mediated allergen-specific immune response [28,34]. This delayed-type hypersensitivity reaction has already been proposed as a pathogenetic mechanism of APC by Dargent et al [35]. It has been suggested that an allergic reaction in the first week of life (as seen in our 7-day-old patient) could be due to an intra-uterine sensitization, secondary to transplacentar antigen passage [14]. The exposure to food antigens in a sensitized infant may lead to hypersensitivity response types I, III and IV (according to Gell and Coombs' classification). Some authors suggested both intestinal immaturity and marked eosinophilic infiltration, which may significantly alter tight junctions, lead to increased intestinal permeability to food proteins [36]. Eosinophils are often clustered in proximity to the lymphoid aggregate below an epithelial erosion, also noted in some of our patients (Figure 2); this observation suggests their role in response to antigen uptake and the possible site of T cell interaction [1]. It is thought today eosinophils may be directly responsible for tissue injury in allergic colitis and it is interesting to note that these cells can bind IgA, which are normally present in breast milk, and undergo degranulation. Eosinophil-derived mediators can stimulate a secretory response from epithelial cells in vitro; this may represent an important pathway in the development of diarrhea. The immature immune system fails to prevent the infiltration of eosinophils, leading to the destruction of epithelial cells, which are responsible for clinical features [6].

## Conclusions

In conclusion, APC induced by food proteins should always be considered in differential diagnosis of hematochezia, especially in exclusively breast-fed infants. Diagnostic endoscopy with biopsies should only be performed in select cases refractory to a maternal hypoallergenic diet. The most appropriate allergological test in delayed gastrointestinal reactions seems to be the APT. We suggest patch testing for foods could become an important diagnostic tool to identify a polisensitization, with the potential objective to correctly address the maternal diet. Patch testing for breast milk seems to be able to demonstrate the passage of food proteins deriving from the maternal diet in exclusively breast-fed infants. We speculate patch testing for foods could become an important diagnostic tool to identify a polisensitization, ever in breast fed infants. In our cases the APT accuracy has been verified by endoscopic resolution after AAF diet instead oral challenge. An AAF should only be prescribed in select cases, especially whether the rectal bleeding is severe as in our case series.

## Abbreviations

APC: allergic proctocolitis; APT: atopy patch test; eHF: extensively hydrolyzed formula; AAF: amino acid-based formula; CM: cow's milk; LNH: lymphoid nodular hyperplasia; HPF: high power field.

## Competing interests

The authors declare that they have no competing interests.

## Authors' contributions

SL: Protocol design, editing manuscript, DN: gastroenterology and endoscopy evaluation, editing manuscript, SF: gastroenterology and endoscopy evaluation, editing manuscript, YD: protocol design, editing manuscript,, GL: protocol design, editing manuscript, TF^1^: protocol design, editing manuscript, AM: tissues processing, histologycal analysis of biopsies, TF^2^: protocol design, allergology evaluation, editing manuscript, SC: protocol design, editing manuscript, All authors have read and approved the final version of manuscript.

## Declaration

All patients received usual care during the course of the study.

## Consent

Written informed consent was obtained from the patient for publication of this case report and any accompanying images. A copy of the written consent is available for review by the Editor-in-Chief of this journal.

## Pre-publication history

The pre-publication history for this paper can be accessed here:

http://www.biomedcentral.com/1471-230X/11/82/prepub
